# 4498 The Effect of the Affordable Care Act on the Stage at Diagnosis in Low income Privately Insured Cancer Patients, including those with Marketplace coverage

**DOI:** 10.1017/cts.2020.433

**Published:** 2020-07-29

**Authors:** Uriel Kim, Siran Koroukian, Johnie Rose

**Affiliations:** 1 Case Western Reserve University

## Abstract

OBJECTIVES/GOALS: The goal of this study was to examine the change in the odds of being diagnosed with metastatic cancer after the Affordable Care Act (ACA) among low-income, privately insured, nonelderly patients with newly diagnosed cancer. Low-income was defined as having income<250% FPL (federal poverty level). METHODS/STUDY POPULATION: Using Ohio cancer registry data linked with census tract-level income data, individuals aged 18-64 years diagnosed with one of the 15 leading cancers and reported being privately insured or uninsured were identified. Low-income patients were isolated using probability weighting, a process in which each observation was assigned a weight equal to the probability of a patient having an income <250% FPL based on the patient’s census tract of residence. Then, a multivariable logistic model was fitted to examine the independent association between the exposure (Post-ACA, years 2015-2016 versus Pre-ACA, years 2012-2013) and the outcome (metastatic versus non-metastatic disease at diagnosis). RESULTS/ANTICIPATED RESULTS: Between the Pre-ACA and Post-ACA periods, the percent uninsured in the low-income study population decreased from 14.1% to 4.5% (p <0.01). In the Post-ACA period, among those with insurance coverage, an estimated 11.7% of individuals had Marketplace coverage. After adjusting for potential confounders (sex, age, race-ethnicity, marital status, community-level income, rurality, and cancer type), individuals diagnosed Post-ACA had 5% lower odds of having metastatic disease relative to Pre-ACA (Adjusted Odds Ratio: 0.95, 95% Confidence Interval: 0.91 - 0.99, p = 0.04). DISCUSSION/SIGNIFICANCE OF IMPACT: The shift towards non-metastatic disease likely reflects increases to coverage brought on by the marketplaces. However, the shift is smaller than those observed in Medicaid enrollees, suggesting that policy refinements in the marketplaces can further improve outcomes in low-income cancer patients.

